# Visual Attention to Food Content on Social Media: An Eye-Tracking Study Among Young Adults

**DOI:** 10.3390/jemr18060069

**Published:** 2025-11-20

**Authors:** Aura Lydia Riswanto, Seieun Kim, Youngsam Ha, Hak-Seon Kim

**Affiliations:** 1 Department of Global Business, Kyungsung University, Busan 48434, Republic of Korea; auralee@ks.ac.kr; 2 Department of Global Hospitality Management, Kyungsung University, Busan 48434, Republic of Korea; celinekim@ks.ac.kr; 3 Department of China Studies, Kyungsung University, Busan 48434, Republic of Korea; ysha@ks.ac.kr; 4 School of Hospitality & Tourism Management, Kyungsung University, Busan 48434, Republic of Korea

**Keywords:** eye-tracking, visual attention, food marketing, social media advertising, purchase intention, young adults, digital food marketing

## Abstract

Social media has become a dominant channel for food marketing, particularly targeting youth through visually engaging and socially embedded content. This study investigates how young adults visually engage with food advertisements on social media and how specific visual and contextual features influence purchase intention. Using eye-tracking technology and survey analysis, data were collected from 35 participants aged 18 to 25. Participants viewed simulated Instagram posts incorporating elements such as food imagery, branding, influencer presence, and social cues. Visual attention was recorded using Tobii Pro Spectrum, and behavioral responses were assessed via post-surveys. A 2 × 2 design varying influencer presence and food type showed that both features significantly increased visual attention. Marketing cues and branding also attracted substantial visual attention. Linear regression revealed that core/non-core content and influencer features were among the strongest predictors of consumer response. The findings underscore the persuasive power of human and social features in digital food advertising. These insights have implications for commercial marketing practices and for understanding how visual and social elements influence youth engagement with food content on digital platforms.

## 1. Introduction

The rise of social media has fundamentally reshaped the landscape of consumer advertising, particularly in the food and beverage sector. In 2024, global social media advertising expenditures are projected to surpass $270 billion, driven by the dominance of highly visual platforms such as Instagram, TikTok, and YouTube [1]. This explosive growth has coincided with increased digital targeting by food brands, which use vibrant visuals, influencer partnerships, and platform-specific trends to engage young audiences [2]. Among these audiences, adolescents and young adults are collectively referred to as “youth” in this study. We define youth as individuals aged 18 to 25, following arguments for an extended period of adolescence [3,4].

Historically, studies on food marketing have focused on traditional media such as television and packaging [5,6]. However, the rapid rise of social media has significantly altered the way youth encounter branded content. As platforms like TikTok and Instagram have become central to daily media consumption, food marketers have redirected their efforts toward digital spaces, particularly through influencer marketing strategies [7,8]. These campaigns often blur the lines between entertainment and advertising, making it difficult for young users to recognize persuasive intent.

Empirical studies show that food brands actively leverage these ecosystems to reach youth. Potvin Kent et al. (2024) [9] found that a significant portion of food marketing targeted at youth on platforms like Instagram and TikTok promoted fast food products, frequently using influencers and viral trends. Vaughan et al. (2025) [10] similarly observed that digital ads are more engaging and memorable to younger users than traditional advertisements, leading to higher brand recall and responsiveness. In light of these trends, the study incorporated fast food examples within the stimuli to better align with the marketing content frequently targeted at young consumers online.

Youth are especially susceptible to digital food marketing due to their ongoing development of critical thinking and media literacy skills, which can hinder their ability to identify and resist persuasive techniques [11]. Food marketing is a major external influence that shapes their food preferences, attitudes, and consumption habits [12]. Despite this, there remains a lack of research specifically examining adolescents’ and young adults’ recall, awareness, and perceptions of such marketing in the digital context.

These developments highlight an urgent need not only to quantify youth exposure to food marketing but also to understand how they visually engage with it. Eye-tracking offers a reliable method for measuring visual attention [13], providing insight into which elements of food advertising capture the gaze and how that attention relates to attitudes and behavioral intentions. This study examines how young adults interact with food content on social media, using eye-tracking methodology to explore the visual and psychological mechanisms that shape consumer responses in digital food marketing.

## 2. Literature Review

### 2.1. Social Media as a Persuasive Advertising Environment

Social media has emerged as a highly immersive and persuasive advertising channel, especially among younger demographics [14]. As of 2020, there were approximately 3.6 billion social media users worldwide, a number expected to grow to 4.4 billion by 2025 [15]. Unlike traditional media, where advertisements are clearly demarcated, social media advertising is integrated directly into user-generated content [16]. Brands can promote products via influencer partnerships, viral challenges, and algorithmically promoted posts, often in ways that appear native to the platform environment [17]. This strategy allows advertising to mimic peer interaction, significantly lowering consumer resistance and increasing perceived credibility [18].

From a psychological perspective, the visual-first design of platforms such as Instagram and TikTok enhances the persuasive power of food-related content [19]. Sensory-rich images, including vivid colors and dynamic food visuals, can activate the reward system, increase visual attention, and stimulate craving responses through enhanced appetitive processing [20]. What makes these ads uniquely influential is their ability to trigger emotional responses and convey useful information, which, when reinforced by likes, comments, and shares, enhances social validation and encourages sharing behavior [21]. Recent studies have also highlighted the role of algorithmic personalization in increasing ad relevance. Based on a user’s browsing behavior, engagement history, and demographic traits, social media platforms deliver content tailored to individual preferences [22]. Additionally, engagement metrics such as follower counts and view numbers act as social proof, reinforcing a sense of credibility and desirability [23].

Importantly, these persuasive features are not limited to overt advertising. Native content, like casual food posts from influencers or aesthetically framed visuals, can exert a subliminal effect [24], influencing attitudes and consumption behaviors even when users are not consciously processing them as ads [25]. These forms of covert persuasion make social media a unique and particularly powerful environment for shaping food choices.

### 2.2. The Food Industry

Over the years, food has shifted from being a basic need to representing culture, identity, creativity, career paths, and social engagement [26]. Reflecting this change, the food industry has also transformed, with globalization and digital technologies redefining how people market, consume, and interact with food in today’s connected world [27]. Traditionally dependent on billboards and TV spots, food brands now rely heavily on social media campaigns that reflect the humor, pace, and aesthetics of contemporary youth culture [28]. This strategic shift enables brands to reach consumers in more personalized, contextually relevant, and visually engaging ways.

In particular, food brands now use platforms popular among young people, such as Instagram, TikTok, and YouTube, to seamlessly integrate advertising into everyday online experiences and entertainment content [29]. This transition has not only increased their visibility but also allowed them to embed brand messaging within the digital habits of users [30]. One of the industry’s most effective tactics is leveraging influencers, both micro and celebrity level, to subtly promote food through storytelling, challenges, or lifestyle vlogs [31]. These representations often disguise marketing as casual sharing, making the content more relatable and persuasive [32,33].

In this rapidly changing digital food landscape, fast food brands have established themselves as especially influential players [34]. The global fast food market is projected to grow steadily from $780.61 billion in 2025, reflecting a total increase of $296.76 billion, which represents approximately 38% growth over the decade [35]. Recent studies have shown that fast food content on social media tends to emphasize non-core foods, such as burgers, fries, and sugary drinks, rather than nutritious alternatives [36]. These posts are typically crafted to convey indulgence and enjoyment, using vibrant visuals, playful text, and time-sensitive deals that encourage immediate sharing and heightened appeal.

Rather than relying solely on traditional advertising, food marketing increasingly utilizes social engagement indicators such as likes, shares, and follower counts, along with algorithm-driven visibility to maintain its presence online [37]. Even brief or incidental exposure to these messages can reinforce brand familiarity and subtly influence consumer preferences over time [38]. The repeated exposure to emotionally engaging and socially relatable content, seamlessly embedded into daily social media use, shapes consumer attitudes and behaviors, often without users consciously recognizing it as advertising [39]. As these strategies continue to evolve within digital platforms, it becomes increasingly important to identify which specific visual and contextual elements are most effective in capturing attention and encouraging engagement [40]. To address this, the present study experimentally evaluates the impact of key features found in real-world food content presented in a simulated social media format.

### 2.3. Visual Attention and the Use of Eye-Tracking in Food Media Research

Eye-tracking has become an essential methodology in food marketing research, providing objective data on how consumers visually engage with different elements of food media [41]. This method tracks metrics such as dwell time, fixation count, and gaze duration, offering valuable insight into attention patterns that may influence decision-making and behavior [42,43]. In food marketing contexts, especially those involving packaging, labels, and branding, eye-tracking allows researchers to isolate which specific visual features prompt cognitive processing or consumer interest [41].

Recent studies have applied eye-tracking to assess consumer responses to sustainability labels, branding elements, and product claims across various food types. For instance, Grebitus et al. (2015) [44] found that organic and environmentally friendly packaging cues increased visual attention, but that this effect was moderated by prior familiarity. Similarly, Conoly and Lee (2023) [45] demonstrated that local origin labels significantly boosted visual attention and influenced food choices. However, not all labels succeed in capturing attention, Babakhani et al. (2020) [46] reported minimal engagement with carbon and farmer labels despite their inclusion in fast food imagery.

Table 1 summarizes key eye-tracking studies relevant to food marketing, illustrating the diversity in stimuli, methodology, and findings. Together, these studies inform our understanding of how visual cues, whether health-related, ethical, or promotional, shape user attention and preference formation.

While the majority of these studies focus on packaging, menu design, or static food labels, relatively few have explored how eye-tracking can be applied to dynamic digital environments like social media. Platforms such as Instagram and TikTok introduce additional variables, such as scrolling behavior, profile icons, like counters, and comments, that may significantly influence gaze behavior and attention allocation. In this context, visual attention is not only influenced by the product itself but also by the social and interactive features embedded in the interface.

By focusing on food imagery within the naturalistic structure of social media feeds, the present study addresses a key gap in the literature. It contributes to our understanding of how youth visually process food marketing in real-time, interactive media spaces where traditional advertising boundaries are blurred. This approach enhances ecological validity and provides insights into how attention to digital marketing cues may influence real-world decision-making and purchase intentions.

## 3. Methodology

In this study, a mixed-method approach was employed to examine visual attention and consumer responses using eye-tracking technology and survey analysis. Data collection was conducted with 35 participants aged between 18 and 25 (Mean age = 23.4 years), using the Tobii Pro Spectrum eye-tracker (1200 Hz) and Tobii Pro Lab software version 24.21. The sample consisted of 20 male (57%) and 15 female (43%), all participants reported normal or corrected-to-normal vision and provided informed consent prior to participation. Participants reported an average of 3.8 h (SD = 1.6) of daily social-media use. The most frequently used platforms were Instagram (94%), TikTok (82%), and YouTube (74%). Participants were exposed to visual stimuli while their eye movements were recorded to generate heatmaps, gazeplots, and fixation metrics, capturing detailed patterns of visual attention.

After the eye-tracking session, the same participants completed a post-survey designed to evaluate changes in perception, attitudes, and purchase intentions based on their visual engagement. Participation was entirely voluntary, and participants were informed that they could decline to participate or withdraw from the study at any time without any consequences.

During the data processing phase, Areas of Interest (AOIs) were defined for each stimulus, and fixation data were refined to isolate relevant metrics. As shown in Table 2, to ensure ecological validity in examining how young adults respond to food imagery, this study bases its stimuli on the coding framework proposed by Qutteina et al. (2019) [6]. By identifying the content and context of food-related posts, the authors created a comprehensive coding scheme that captures both visual and social features of digital food content.

**Figure 1:**
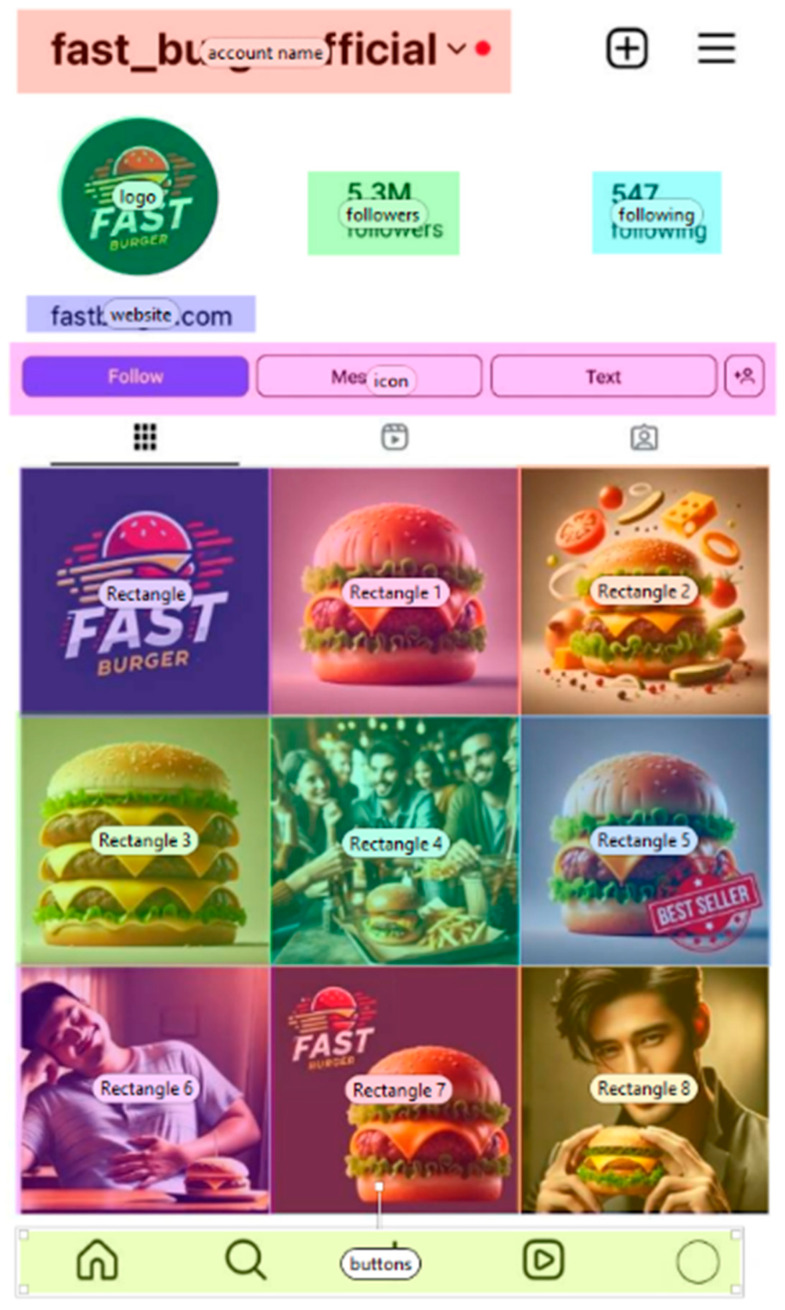
Instagram Stimulus (Instagram mobile UI, app v385.0) with Marked AOIs (Areas of Interest).

The Instagram stimulus used in the experiment displays a simulated food brand profile, as shown in Figure 1. The numbered rectangles in Figure 1 (e.g., Rectangle 1, Rectangle 2) represent the predefined Areas of Interest (AOIs) outlined in Table 2. While the visual labels remain generic, each rectangle was mapped to a specific AOI during the analysis. For example, Rectangle 1 corresponds to “food depicted”, Rectangle 2 to “core/non-core”, Rectangle 3 to “quantity”, and so on. Each Instagram-style post depicted a single focal food item. The AOI for number of followers was defined based on the follower count section in the profile. This alignment between the visual stimulus and the coding framework ensured consistent measurement of gaze behavior across distinct content features, enabling a detailed assessment of how specific visual elements influenced attention and response. To minimize exogenous cueing, mock-ups contained no arrow or chevron icons, pointing graphics, or other directional symbols that could elicit automatic attention orienting. Layout and object placement were held symmetric across conditions [48].

To ensure the internal validity of our analysis and allow for the isolation of key content effects, we implemented a 2 × 2 experimental design manipulating influencer presence (present vs. absent) and food type (core vs. non-core). Consistent with youth food-marketing classifications summarized by Qutteina et al. (2019) [6], we adopt the “core vs. non-core (discretionary)” taxonomy but implement it as a visual framing proxy suitable for image-based stimuli. Posts were coded core-framed when the depiction foregrounded whole-food ingredients (e.g., visible vegetables/salad elements) and a standard single portion without prominent cheese/sauce overlays; posts were coded non-core-framed when the depiction emphasized indulgent cues (e.g., layered cheese/sauces, multiple patties, oversized portions).

All stimuli were presented in a randomized order for each participant to minimize sequence effects, reduce bias, and enhance the reliability of gaze and behavioral measurements. All mock-ups replicated the Instagram mobile interface (app v385.0, Meta Platforms, Inc.) while holding constant the global layout (header, 3 × 3 post grid, buttons), image size and position, background, color balance, and the placement of branding and follower metrics. Screens were presented full-screen on a desktop LCD monitor attached to the Tobii Pro Spectrum (1200 Hz), with participants seated at a comfortable viewing distance as per Tobii Pro Lab guidelines. Stimuli were static and non-interactive; interface buttons could not be clicked.

**Figure 2:**
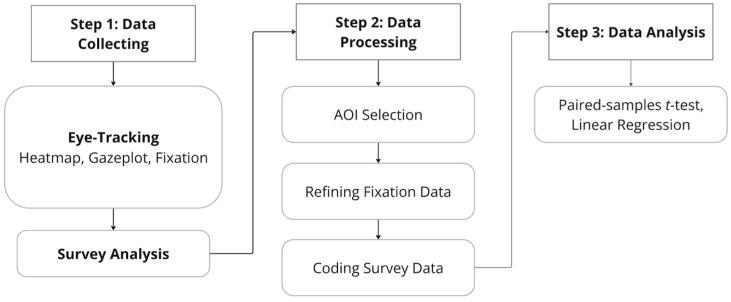
Overview of the Research Process.

Following the eye-tracking session, the data processing and analysis stages involved integrating both visual and survey-based measures, as illustrated in Figure 2. Survey responses from both pre- and post-surveys were systematically coded to align with key variables of interest. The post-exposure survey assessed purchase intention using a single 5-point Likert item (1 = strongly disagree; 5 = strongly agree): “I would consider purchasing the item shown.” Higher scores indicate stronger intention to purchase.

For the final analysis, paired-samples *t*-tests were used to assess differences in visual attention and attitudinal shifts, while linear regression analyses were conducted to explore the predictive relationship between fixation patterns and consumer responses. This integrated methodology allowed for a comprehensive investigation of how visual elements influence attention, perception, and decision-making among young adult consumers.

## 4. Results

**Figure 3:**
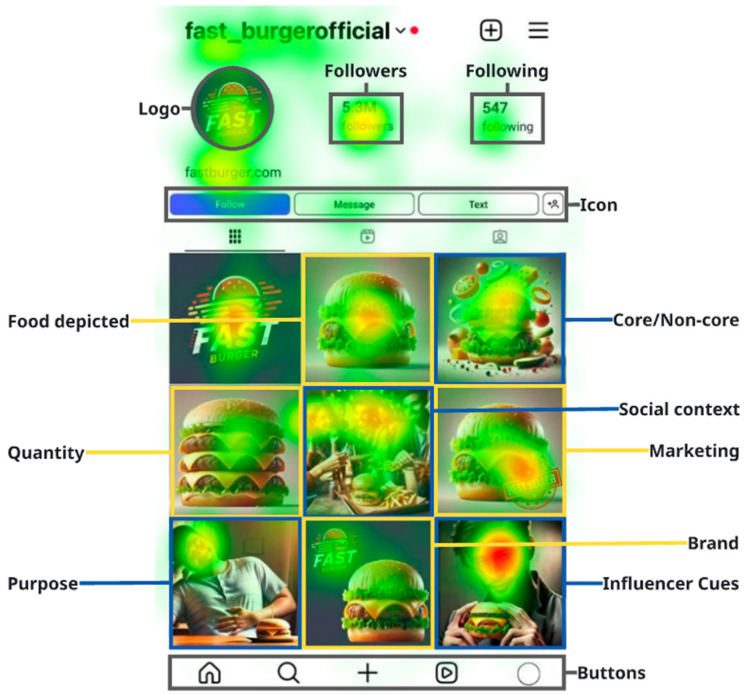
Heatmap Visualization Results.

Figure 3 displays an eye-tracking heatmap of a branded Instagram profile, illustrating participants’ visual attention across predefined interface elements. Thirty-five participants (20 males, 57%; 15 females, 43%) aged 18–25 years (M = 23.4) were included. Participant-level fixation heatmaps were generated in Tobii Pro Lab using duration-weighted fixations with Gaussian smoothing. For each stimulus, heatmaps were averaged across all participants to obtain a group-level gaze density map, and for all AOIs, attention was summarized as dwell time (seconds), defined as the sum of fixation durations within an AOI. For visualization, values were min–max normalized within stimulus; warmer colors indicate higher fixation density [49]. As expected, the heatmap indicates that participants’ gaze was concentrated on central visual features [50], including food images, branding elements (e.g., profile logo and icon), and human faces within the content.

Table 3 presents the descriptive statistics derived from the eye-tracking analysis of five key interface elements commonly found on social media platforms: buttons, followers, following, icon, and logo. The accompanying heatmap uses red to indicate higher values, yellow intermediate values, and green lower values. The metrics include the mean fixation duration, standard deviation, and standard error of the mean for each element. The logo (*M* = 0.4589, *SD* = 0.32394) and followers (*M* = 0.6631, *SD* = 0.32903) elements recorded the highest mean fixation durations, suggesting that these components attracted the greatest visual attention among users. In contrast, buttons showed the lowest average attention (*M* = 0.1350, *SD* = 0.02121), indicating limited visual engagement.

Table 4 presents descriptive statistics of eye-tracking analysis measuring visual attention to various types of food-related social media content. Each category represents a commonly observed feature in food imagery, including the type of food shown, social cues, branding, and promotional elements. The results indicate that marketing-related content attracted the highest visual attention (*M* = 4.00, *SD* = 0.804), highlighting the salience of promotional elements such as slogans, discount offers, and product claims.

Additionally, in content featuring people, whether in social contexts (*M* = 3.57) or posted by influencers (*M* = 3.29), also drew substantial attention. These categories included visual depictions of individuals, such as friends dining together or renowned figures.

Linear regression was conducted to examine which visual elements significantly predict purchase intention as shown in Table 5. The model included nine predictors: food depicted, core/non-core classification, quantity, social context, marketing, purpose, influencers, brand, and number of followers. The overall regression model was statistically significant, *F* = 2.27, *p* = 0.045, and explained approximately 45.0% of the variance in purchase intention (*R*^2^ = 0.450, Adjusted *R*^2^ = 0.370).

Among the predictors, Core/Non-core (*β* = 0.624, *p* = 0.001), Social Context (*β* = 0.398, *p* = 0.007), Influencers (*β* = 0.379, *p* = 0.017), and Number of Followers (*β* = 0.343, *p* = 0.005) significantly contributed to the model. Other variables such as Marketing (*β* = 0.291, *p* = 0.050) showed marginal significance, while predictors like food depicted, quantity, purpose, and brand were not significant.

To further examine the effects of specific content features, additional analyses were conducted focusing on two experimentally manipulated variables: influencer presence and food type (core vs. non-core). These variables were systematically varied across the stimuli to enable isolated comparisons. The results of the linear regression analysis (Table 6) showed that both variables had statistically significant effects on visual attention, with core/non-core status (*B* = 0.758, *p* = 0.001) and influencer presence (*B* = 0.477, *p* = 0.017) emerging as strong predictors. These findings support the notion that controlled variation of key features can yield meaningful insights into viewer responses.

As shown in Table 6, both food type and influencer presence had statistically significant effects on visual attention. Specifically, core food content (*B* = 0.740, *p* = 0.001) and the presence of an influencer (*B* = 0.510, *p* = 0.013) significantly increased participants’ visual attention. The overall model was significant, *F*(2, 97) = 18.24, *p* < 0.001, explaining 41.2% of the variance in visual attention (Adjusted *R*^2^ = 0.388).

## 5. Discussion

This study examined how young adults visually engage with food content on social media and how specific content characteristics influence their purchase intentions. These effects were identified through a controlled experimental design, which allowed for the isolation of influencer presence and food type, strengthening the causal interpretation of the findings. Using eye-tracking methodology and survey data, the study identified several content features, namely, the core/non-core classification of food, social context, influencer presence, and follower count, as significant predictors of visual attention and behavioral intention. By integrating the post-exposure purchase-intention measure with fixation-based AOI attention, we move beyond describing where participants look to clarifying what they are likely to do, yielding a more holistic understanding of visual attention in digital food advertising. The prominence of the core/non-core food distinction as a predictor suggests that even in the context of food marketing, nutritional framing can meaningfully influence consumer responses.

The influence of social context and influencer presence supports previous research emphasizing the importance of human cues in digital advertising. De Veirman et al. (2017) [51] showed that influencer marketing enhances engagement and brand trust, particularly when the content appears authentic or peer-driven. Similarly, Bindemann et al. (2005) [52] found that human faces tend to capture and hold visual attention more effectively than non-human elements. Min et al. (2017) [53] further demonstrated that facial features can be used to predict visual attention patterns. This finding aligns with Nazzaro et al. (2025) [54], who reported that consumers are more attentive to food content emphasizing health or functional value. Although fast-food marketing is often associated with indulgent, non-core items, the present results suggest that young adults still attend to visual cues linked to healthier or more balanced food options.

The significance of follower count as a predictor further illustrates the role of social validation in digital marketing. This is consistent with the findings of Naylor et al. (2012) [55], who argued that higher social media metrics contribute to perceived brand credibility and increase consumer willingness to engage. In the present study, the attention given to follower metrics suggests that users may use such indicators as heuristics for trustworthiness or popularity, which in turn influence their behavioral responses. Compared to earlier studies that focused on food packaging, menus, or static advertisements [44,46] the present study offers a more ecologically valid approach by simulating static, non-interactive Instagram-style posts within a platform-native, interface-embedded feed that retains key social cues (profile icon, captions, follower metrics, and action buttons). The integration of interface elements such as profile icons, buttons, and follower statistics reflects the multifaceted nature of digital consumption and allows for a more comprehensive analysis of visual behavior. Beyond individual attention and intention, platform-level responsibility for food health can also be advanced by automated image analysis. Recent work on zero-shot, knowledge-enhanced detection of visual food-safety hazards demonstrates scalable identification of previously unseen contamination cues without retraining, suggesting how algorithmic screening could complement gaze-based evidence to prioritize moderation or health messaging in social feeds [56].

This study was intentionally scoped to young adults (18–25), the cohort most engaged with visual-first platforms. Within this target population, the sample was modest (N = 35) and drawn from a single university in one country, which may limit representativeness across institutions, regions, and socioeconomic backgrounds. Compared to prior eye-tracking studies in the food marketing domain, the sample in this study is modest. For instance, van Loo et al. (2021) [57] conducted an experiment involving 115 participants to assess the effects of nutrition and sustainability claims on granola bar preferences. Similarly, Oselinsky et al. (2021) [58] employed a sample of 434 participants in their investigation of GMO food labeling and college student selection behavior. Rihn and Yue (2016) [59], for instance, conducted a study exploring visual attention and willingness-to-pay for processed food, recruited 93 participants. These examples illustrate that larger sample sizes are often used in comparable research, which enhances statistical power and external validity. Future studies should aim to recruit broader and more diverse populations to validate and extend the present findings. Participants’ gender distribution, academic background, and social media use patterns may also influence attention and purchase intentions. As these factors were not controlled for, they represent potential confounders. Future studies with larger and more diverse samples should include these variables as covariates to more precisely isolate the effects of content features.

## 6. Conclusions

This study investigated how specific visual and contextual features of food marketing on social media influence young adults’ visual attention and purchase intentions. Using eye-tracking technology and survey analysis, the results demonstrate that core food content, social context, influencer presence, and follower count significantly affect both visual engagement and consumer intention. This study used a systematically manipulated experimental design to examine how key visual features, specifically, influencer presence and food type, affect visual attention in food advertisements on social media. These findings provide evidence that digital food marketing is shaped not only by product imagery but also by social cues embedded within platform interfaces.

From a marketing perspective, the results underscore the importance of incorporating human-centered and socially validated design elements to enhance the effectiveness of food advertising. Features such as the presence of influencers or prominent follower counts can significantly increase consumer engagement, even in brief exposures. These insights clarify how visual and social elements shape youth engagement with food content on digital platforms and may inform media-literacy efforts, public communication, and regulatory guidance.

From a public health standpoint, the findings raise important considerations regarding the subtle influence of social media-based food marketing. Given that even minor nutritional cues, such as the representation of core food components, can affect purchase intention, there may be opportunities to apply similar persuasive strategies in promoting healthier food options. However, the capacity of emotionally resonant and socially embedded content to shape consumer behavior also calls for further attention from policymakers and health communicators, particularly regarding youth exposure to unhealthy food messaging. These implications align with strategic policy roadmaps such as Food Vision 2030, which emphasize healthier food environments and responsible digital marketing [60]. Complementary design research advocates evidence-based, human-centered approaches to counter influencer-driven nutrition misinformation in social media ecosystems [61].

Despite these contributions, the study’s limitations, particularly its small and demographically narrow sample, should be addressed in future research. Larger and more diverse participant groups, as well as the inclusion of behavioral outcome measures such as actual purchasing behavior, would provide a more robust understanding of how visual attention translates into real-world decision-making. Given that inhibition of return (IOR) effects were not explicitly modeled, any such effects likely introduced nonsystematic variance; future work should incorporate IOR-sensitive scanpath models and spatial counterbalancing to better dissociate content from spatial-memory influences [62]. Given that most prior work has been conducted in controlled, lab-based settings [63], future studies should extend these paradigms by pairing eye-tracking with complementary physiological indices, heart-rate variability, EEG, and pupillometry, to more comprehensively characterize attentional and affective states during exposure [64].

In conclusion, the study provides meaningful insights into the mechanisms by which social media food marketing engages young consumers. It highlights the need for further interdisciplinary research that bridges marketing, psychology, and public health to better understand and influence food choices in the digital age.

## Figures and Tables

**Table 1:** Summary of Eye-Tracking Research on Food Media Stimuli.

Author (Year)	Country	Food Type	Sample	Stimuli	Eye-Tracker	Measure	Key Findings
Babakhani et al. (2020) [46]	Australia	Burgers, drinks, desserts	54, avg. 32 yrs, 62% female	Carbon & local farmer labels	Tobii TX-300 (300 Hz)	Dwell time, time to first fixation	Labels captured little attention and did not influence menu choice
Balcombe et al. (2017) [47]	UK	Pepperoni pizza	100, varied ages	Organic & country-of-origin labels	EyeLink II (500 Hz)	Dwell time, fixation count	Sustainable preferences led to more attention on such labels
Conoly & Lee (2023) [45]	USA	Menu choice	50, avg. 30.8 yrs, 52% female	Local label	Tobii X2-60 (60 Hz)	Fixation count, duration	The extrinsic increased visual attention and influenced choice
Grebitus et al. (2015) [44]	USA	Cheddar cheese	130, younger, educated	Hormone-free, origin, biodegradable packaging	Tobii T60 XL (60 Hz)	Dwell time	Visual attention increases with organic labels but depends on familiarity

**Table 2:** The Area(s) of Interest (AOIs) defined for this study.

AOI	Description
Food depicted	The specific food item displayed in the post.
Core/Non-core	Whether the item aligns with dietary guideline food groups (core) or is discretionary (non-core).
Quantity	Serving size shown relative to a standard portion (regular vs. excessive).
Social context	Presence of people or social scenes (e.g., friends, family, celebrations).
Marketing	Visible promotional elements (e.g., slogans, discounts, price tags, sales claims).
Purpose	Apparent intent of the post (lifestyle/artistic sharing vs. explicit promotion).
Brand	Visibility of brand identifiers (logo, brand name, recognizable packaging).
Influencer Cues	Source/account type (everyday user, micro-influencer, celebrity influencer).
Followers	Follower-count region in the profile header indicating potential reach.

**Table 3:** Dwell time on content AOIs.

Element	Mean	Standard Deviation	Standard Error Mean
Buttons	0.135	0.021	0.015
Followers	0.663	0.329	0.082
Following	0.380	0.240	0.060
Icon	0.430	0.405	0.093
Logo	0.458	0.323	0.061

Notes: Content AOIs: mean dwell time (s) per participant per stimulus.

**Table 4:** Visual Attention Metrics by Type of Food-Related Content on Social Media.

Content Type	Mean	Standard Deviation	Standard Error Mean
Food depicted	3.40	0.775	0.131
Core/Non-core	3.71	0.957	0.162
Quantity	3.60	0.881	0.149
Social context	3.57	0.948	0.160
Marketing	4.00	0.804	0.136
Purpose	3.26	1.120	0.189
Influencers	3.29	1.100	0.186
Brand	3.94	0.725	0.123

Notes: Content AOIs: mean dwell time (s) per participant per stimulus.

**Table 5:** Linear Regression Results.

Predictor	B	Std. Error	Beta	*t*	Sig.
(Constant)	2.102	0.547		3.844	<0.001 ***
Food depicted	0.083	0.178	0.071	0.466	0.642
Core/Non-core	0.758	0.215	0.624	3.528	0.001 ***
Quantity	−0.289	0.249	−0.194	−1.161	0.248
Social Context	0.402	0.140	0.398	2.871	0.007 **
Marketing	0.338	0.166	0.291	2.036	0.050
Purpose	−0.123	0.228	−0.089	−0.539	0.591
Influencers	0.477	0.188	0.379	2.540	0.017 *
Brand	0.209	0.312	0.114	0.670	0.508
Number of followers	0.585	0.189	0.343	3.096	0.005 **

Notes: Dependent Variable: Purchase intention from social media; *R*^2^ = 0.450; adjusted *R*^2^ = 0.370; *F* = 2.27, *p* < 0.05 (*), *p* < 0.01 (**), *p* < 0.001 (***).

**Table 6:** Linear Regression Predicting Visual Attention from Food Type and Influencer Presence.

Predictor	B	Std. Error	Beta	*t*	Sig.
(Constant)	2.450	0.402		6.096	<0.001 ***
Food Type (1 = Core)	0.740	0.210	0.615	3.524	<0.001 ***
Influencers Presence (1 = Present)	0.510	0.200	0.395	2.550	0.013 *

Notes: Dependent Variable: Visual Attention; *R*^2^ = 0.412; adjusted *R*^2^ = 0.388; *F* = 18.24; *p* < 0.05 (*), *p* < 0.001 (***).

## Data Availability

The data presented in this study are available on request from the corresponding author. The data are not publicly available due to privacy.
